# Practical insights for the clinical implementation of the EULAR recommendations for patients with systemic lupus erythematosus

**DOI:** 10.1136/rmdopen-2025-006210

**Published:** 2025-12-31

**Authors:** Ioannis Parodis, Antonis Fanouriakis, Alessandra Bortoluzzi, Christof Iking-Konert, Julia Weinmann-Menke, Carmen San Román, Roger Abramino Levy, Inigo Rua-Figueroa, Tobias Alexander, Zahir Amoura

**Affiliations:** 1 Division of Rheumatology, Department of Medicine Solna, Karolinska Institutet, Karolinska University Hospital, and Center for Molecular Medicine (CMM), Stockholm, Sweden; 2 Department of Rheumatology, Faculty of Medicine and Health, Örebro University, Örebro, Sweden; 3 Rheumatology and Clinical Immunology Unit, 4th Department of Internal Medicine, Attikon University Hospital, Joint Academic Rheumatology Program, National and Kapodistrian University of Athens School of Medicine, Athens, Greece; 4 Rheumatology Unit, Department of Medical Sciences, University of Ferrara and Azienda Ospedaliero-Universitaria S.Anna, Ferrara, Italy; 5 Department of Rheumatology, Stadtspital Zürich, Zürich, Switzerland; 6 University Medical Center, Department of Nephrology, Rheumatology and Center of Immunotherapy, Mainz, Germany; 7 Respiratory, Immunology and Inflammation Unit, Europe Medical Affairs, GSK, Madrid, Spain; 8 Respiratory, Immunology and Inflammation Unit, Global Medical Affairs, GSK, Collegeville, Pennsylvania, USA; 9 Division of Rheumatology, Hospital Universitario Doctor Negrín, Las Palmas de Gran Canaria, Spain; 10 Charité—Universitätsmedizin Berlin, corporate member of Freie Universität Berlin, Humboldt-Universität zu Berlin and Berlin Institute of Health, Department of Rheumatology and Clinical Immunology, Berlin, Germany; 11 German Rheumatism Research Centre (DRFZ) Berlin, a Leibniz Institute, Berlin, Germany; 12 Sorbonne Université, Inserm, Centre d'Immunologie et des Maladies Infectieuses-Paris (CIMI PARIS), INSERM U1135, Paris, France; 13 Sorbonne Université, AP-HP, Groupement Hospitalier Pitié–Salpêtrière, Centre national de référence du lupus systémique, du syndrome des antiphospholipides et autres maladies auto-immunes, Service de Médecine Interne 2, Institut E3M, Paris, France

**Keywords:** Lupus Erythematosus, Systemic, Glucocorticoids, Biological Therapy, Lupus Nephritis, Treatment

## Abstract

**Objectives:**

The European Alliance of Associations for Rheumatology (EULAR) recommendations for the management of systemic lupus erythematosus (SLE) and lupus nephritis provide important guidance for practitioners on diagnosis, monitoring and treatment. However, practical barriers, such as time constraints, may pose challenges to practitioners when implementing these recommendations in real-world settings. We provide practical, expert-driven advice on how practitioners may effectively and efficiently implement the EULAR recommendations in routine clinical practice.

**Methods:**

Eight international SLE experts convened and contributed opinions and advice for practitioners via an online survey containing 17 open-ended questions on implementation of the EULAR recommendations for early diagnosis, treatment targets and the use of glucocorticoids (GCs), immunosuppressants and biologics. Survey results were compiled and analysed to reach consensus on key advice points for each topic.

**Results:**

Expert advice covered four key topics—setting standardised targets to help modify disease course and prevent organ damage; taking action to achieve these targets; monitoring of target achievement through validated clinical tools and frameworks; and optimising the therapeutic strategy to prevent flares and GC-associated toxicities. A total of 13 core expert-driven advice points were developed across these topics, including scenarios for consideration of earlier biological and/or conventional immunosuppressive use, specific risk factors for poorer prognosis to inform treatment decisions and suggestions on GC tapering.

**Conclusions:**

These expert insights could facilitate implementation of the EULAR recommendations for the management of SLE in clinical practice, thereby helping patients achieve treatment targets and prevent and/or delay organ damage progression.

**WHAT IS ALREADY KNOWN ON THIS TOPIC**

- A European Alliance of Associations for Rheumatology (EULAR) taskforce published updated recommendations for the management of systemic lupus erythematosus (SLE) in late 2023.
- EULAR announced their latest recommendations for lupus nephritis (LN) in 2025.
- Practical barriers may pose challenges to practitioners in the implementation of these recommendations in real-world clinics.

**WHAT THIS STUDY ADDS**

- We provide 13 key advice points, developed by a panel of eight international SLE experts, aimed to help practitioners implement the EULAR recommendations for the management of SLE.
- The advice includes specific scenarios for the consideration of earlier use of targeted biologics and/or conventional immunosuppressants, specific risk factors for poorer prognosis, strategies to help practitioners limit glucocorticoid use and how to facilitate management of severe SLE manifestations, such as LN.

**HOW THIS STUDY MIGHT AFFECT RESEARCH, PRACTICE OR POLICY**

- This practical advice will help practitioners to effectively implement the EULAR recommendations in real-world settings, thereby giving patients with SLE better opportunities for positive outcomes.

## Plain language summary

 The European Alliance of Associations for Rheumatology (EULAR) recommendations for systemic lupus erythematosus (SLE) provide a valuable framework for managing this complex disease. However, practitioners often face obstacles when applying these recommendations in routine care, such as limited time during patient appointments. To help practitioners overcome these challenges, eight international SLE experts came together to provide practical advice on effectively implementing the EULAR recommendations. They identified key concepts through an online survey and developed 13 key points of advice for practitioners across four main areas:

1. Defining clear treatment goals to change the course of the disease and prevent organ damage.
2. Practical strategies for achieving these goals in everyday clinical care.
3. Methods for accurately monitoring patient progress and finding out if medications are working.
4. Optimising treatment plans to reduce medication side effects and prevent flares.

The expert advice also highlights when practitioners should consider earlier use of biologics, how to identify risk factors for poor prognosis and how/when to safely reduce steroid use. Together, these advice points will provide practitioners with a framework to effectively apply the EULAR recommendations for SLE in their daily clinic. Specific recommendations from EULAR on how to treat patients with lupus nephritis (LN) were released in 2025.

## Introduction

SLE is a complex disease that poses challenges in management due to its variable manifestations, treatment-associated toxicities and a fluctuating disease course characterised by flares.^1 2^ The disease heterogeneity also adds challenges for the design of randomised controlled trials when evaluating novel SLE treatments.^2^ Despite an evolving SLE treatment landscape with the emergence of novel treatment classes, such as biologics,^2^ patients with SLE continue to experience detrimental impacts on their quality of life, physical and mental health, as well as a substantial economic burden.^3 4^ Earlier effective intervention is needed to modify the disease course and improve patient outcomes.^5^ A 2024 evaluation identified hydroxychloroquine and belimumab as having sufficient data available to meet the criteria for disease modification in non-renal SLE^6^; other treatments could meet the criteria for disease modification as further evidence accumulates. Several frameworks have been developed to help practitioners achieve positive outcomes for patients, including a framework to implement treat-to-target (T2T) in routine clinical care.^7^

A EULAR taskforce published updated recommendations for the management of SLE and LN in late 2023,^8^ with further LN-specific recommendations published in late 2025.^9^ The recommendations for SLE aimed to provide an up-to-date framework for practitioners across several key areas, such as SLE disease monitoring, pharmacological and non-pharmacological interventions and treatment of specific manifestations and comorbidities.^8^ This notwithstanding, practitioners continue to face challenges to their implementation in daily practice. Potential barriers include uncertainty on the optimal timing of treatment changes, such as when to start a biological or begin glucocorticoid (GC) tapering, lack of time and resources, inexperience with SLE treatment, doubts about selecting the right treatment for the right patient given the wide range of therapeutic options available and a lack of clarity on how to better use monitoring tools to inform treatment plans. Facilitating implementation of the EULAR recommendations is especially vital following the paradigm shift in both SLE and LN towards the importance of earlier effective immunosuppression (with biologics and/or conventional immunosuppressants (ISs)).^8 10 11^ In LN, initial multiagent therapy is recommended, comprising a conventional IS, a biological or calcineurin inhibitor and GCs.^9 11^ To this end, this manuscript aims to provide expert advice and opinions that will help implement the EULAR recommendations in daily practice.

## Methods

Eight renowned international SLE experts from seven European countries convened and contributed opinions and advice for practitioners on the real-world implementation of the current EULAR recommendations for the management of SLE.^8^ Experts were selected to ensure wide geographical representation across Europe, with consideration given to experts who have published in high impact international journals as an author and/or editor, who are an active member of an international or national scientific society, and who have more than 10 years of experience in managing patients with SLE. The experts completed an online survey containing 17 open-ended questions on the implementation of the EULAR recommendations on early diagnosis, monitoring, T2T and the use of GCs, ISs and biologics (see online supplemental materials for full survey questions).

Survey results were compiled and analysed by the experts during an online teleconference workshop to reach consensus on the key advice and practice points for each topic, with further refinement via email discussions prior to the development of this manuscript. The two GSK authors contributed by facilitating discussions among the rest of the authors and providing supporting evidence that reinforced expert opinions. Their involvement was grounded in extensive scientific expertise in autoimmune diseases, particularly in SLE and LN.

## Results

**Figure 1:**
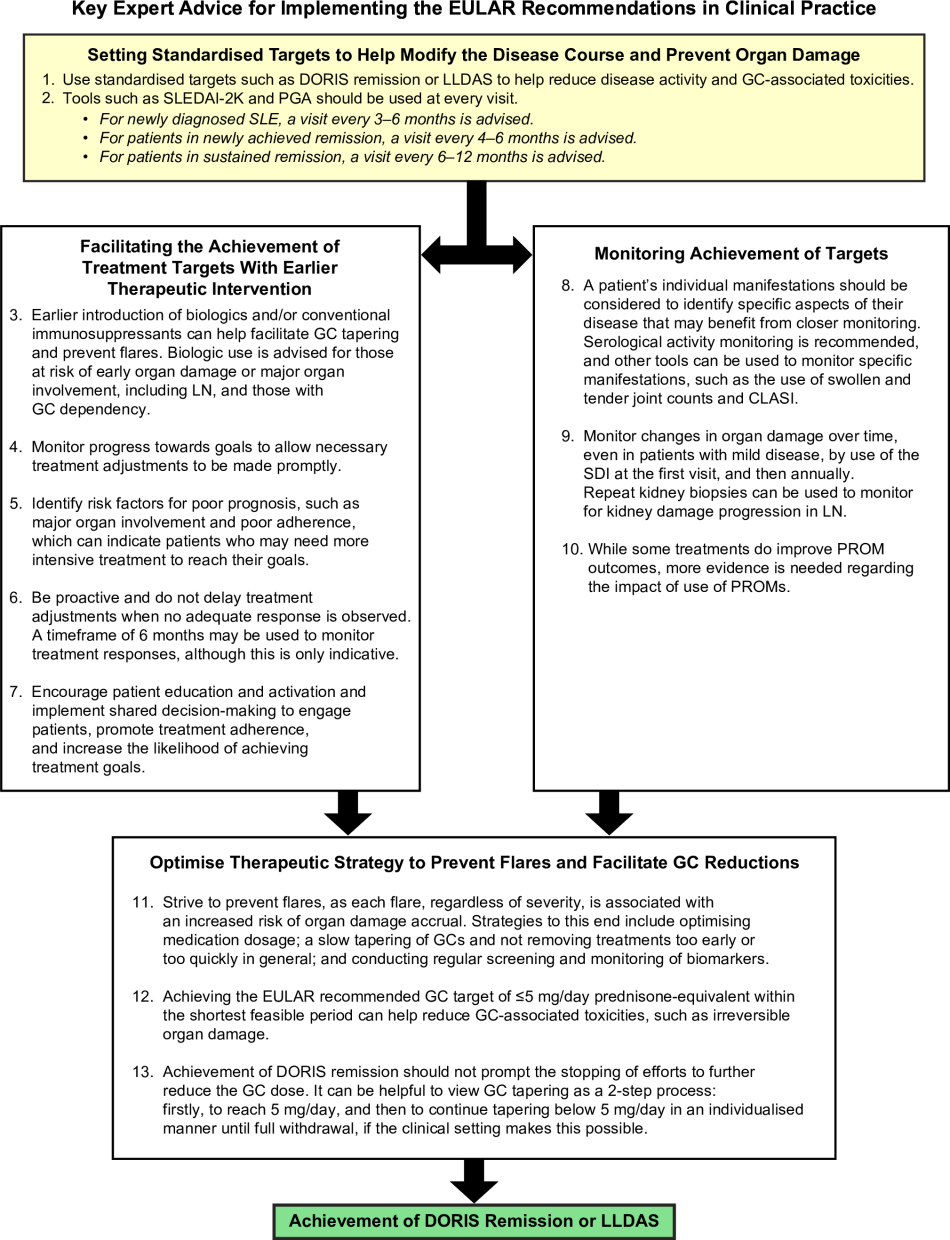
Summary of expert advice for implementing the EULAR recommendations for SLE in clinical practice. CLASI, Cutaneous Lupus Erythematosus Disease Area and Severity Index; DORIS, Definition of Remission in SLE; EULAR, European Alliance of Associations for Rheumatology; GC, glucocorticoids; LLDAS, Lupus Low Disease Activity State; LN, lupus nephritis; PGA, Physician Global Assessment; PROM, patient-reported outcome measure; SDI, Systemic Lupus International Collaborating Clinics/American College of Rheumatology Damage Index; SLE, systemic lupus erythematosus; SLEDAI-2K, Systemic Lupus Erythematosus Disease Activity Index 2000.

Expert opinions on implementation of the EULAR recommendations covered 13 advice points across four key areas (figure 1)—(1) setting standardised targets to help modify the course of the disease and prevent organ damage; (2) earlier therapeutic intervention to achieve these targets (ie, T2T strategy); (3) monitoring of target achievement through validated clinical tools and frameworks; and (4) optimising the therapeutic strategy to prevent flares while facilitating GC dose reductions.

### Setting standardised targets to help modify the disease course and prevent organ damage

**Advice point 1. It is important to aim for achievement of standardised targets over time, such as remission according to the Definition Of Remission In SLE (DORIS) 2021, or low disease activity according to the Lupus Low Disease Activity State (LLDAS) criteria (yet with a lower threshold for GCs of ≤5 mg/day, as according to DORIS).**

(1.1) The EULAR taskforce recommends pursuing a treatment target, ideally remission defined by the DORIS criteria or a state of low disease activity (such as LLDAS; box 1).^812^,^14^ When opting for a low disease activity target, experts are advised to use a GC threshold of ≤5 mg/day prednisone-equivalent (such as LLDAS-5), consistent with the GC threshold of DORIS remission,^14 15^ rather than the standard LLDAS threshold of ≤7.5 mg/day. Achievement of DORIS remission or LLDAS can lower a patient’s risk of organ damage^8^ and thereby help to modify the course of the disease, with SLE disease modification indicated by delayed or prevented damage at >5 years.^5 6^

Box 1 **Definitions of Definition Of Remission In Systemic lupus erythematosus (DORIS) remission and Lupus Low Disease Activity State (LLDAS)**

**DORIS remission^14^**

- Clinical Disease Activity Index (SLEDAI)=0.
- Physician Global Assessment (PGA) <0.5 (0–3 scale).
- Irrespective of serology/no serology negativity requirement (anti-double-stranded DNA, complement).
- Glucocorticoid (GC) ≤5 mg/day.
- Stable antimalarials, immunosuppressants or biologics permitted.

**LLDAS^12^**

- SLEDAI-2K ≤4 with no major organ involvement (renal, central nervous system, cardiopulmonary, vasculitis, fever and no haemolytic anaemia or gastrointestinal activity).
- PGA ≤1 (0–3 scale).
- No new features of lupus disease activity.
- GC ≤7.5 mg/day.
- Standard maintenance doses of immunosuppressants or approved biologics permitted.

(1.2) In their clinical practice experience, experts reported that they observe DORIS remission rates of 25–50%, and LLDAS achievement rates of 35–80%, with LLDAS being a more achievable target. However, even after target achievement, practitioners should aim for further GC reductions towards eventual full withdrawal, per EULAR’s recommendation^8^ (see **Advice points 12–13**).

(1.3) When setting appropriate targets, discussion with the patient is important to set expectations.

(1.4) The optimal time to achieve a DORIS remission or LLDAS target after diagnosis or a flare is not well defined and can vary depending on a patient’s individual manifestations and disease course.

(1.5) Experts emphasised that an adequate treatment response and reaching DORIS remission or LLDAS are not necessarily achieved at the same time. For example, a patient may be considered to show an adequate treatment response, such as a ≥25% reduction in proteinuria in LN within the first 3 months of treatment, even if they do not yet meet the DORIS remission or LLDAS definitions.

(1.6) In LN, the target should be preservation or improvement of kidney function within 3 months, accompanied by a ≥25% improvement in proteinuria by 3 months and ≥50% by 6 months, and a urine protein/creatinine ratio <700 mg/g by 12 months and as low as possible after 12 months, per the EULAR 2025 LN recommendations.^9^ Renin-angiotensin system inhibitors should be considered as supportive therapy for patients with elevated proteinuria, per LN and chronic kidney disease treatment recommendations.^11 16^

**Advice point 2. Although there are often time constraints at patient visits, tools such as the SLE Disease Activity Index 2000 (SLEDAI-2K) and Physician Global Assessment (PGA) should be used at every visit to ensure that practitioners have an accurate estimation of a patient’s disease activity. Visits should be scheduled at least every 3–6 months following SLE diagnosis, or for those with LN or other severe organ involvement, at least every 1–3 months in the initial phase. For patients in newly achieved remission, a visit every 4–6 months is advised, while for patients in sustained remission, a visit every 6–12 months is advised.**

(2.1) Practitioners should also consider monitoring of non-inflammatory symptoms, such as fatigue that is not related to inflammatory disease activity. Awareness of the patient’s non-inflammatory symptoms can help to avoid immunological overtreatment of symptoms that are not directly attributed to inflammation or SLE disease mechanisms.

### Facilitating the achievement of treatment targets through earlier therapeutic intervention

**Advice point 3. Earlier introduction of biologics (ie, belimumab or anifrolumab) and/or conventional ISs should be considered in patients not responding to hydroxychloroquine or unable to reduce their GC dose to acceptable levels, with DORIS remission or low disease activity as the standardised goals.**^8 10^ **For active LN, multiagent therapy with biologics or calcineurin inhibitors (CNIs) in addition to standard immunosuppression is now recommended for all patients.**^8 9 11^

(3.1) While a wide range of patients might be appropriate candidates for earlier biological treatment under the current EULAR recommendations, the experts appreciate that this does not always occur in practice. Experts noted several specific indicators or scenarios in which they would strongly advise considering earlier biological use, although the absence of such indicators does not preclude their earlier use.

First, the presence of moderate-to-high disease activity and a risk of early organ damage accrual or major organ involvement are indicators for consideration of earlier biological use with or without conventional IS, especially for patients with newly diagnosed SLE or a short duration of disease (such as <6 months). For patients with LN or other organ-threatening or life-threatening disease, initial intensive multiagent therapy consisting of GC, a conventional IS and a biological is preferred per the latest international recommendations.^8^,^11^

Finally, for patients with a longer duration of disease, biologics and/or conventional ISs should be considered, particularly for those with an ongoing GC dependency (such as those unable to reduce and sustain doses of ≤5 mg/day prednisone-equivalent; see Advice point 12.1), at risk of new flares or lack of a response to hydroxychloroquine (with or without GC). Baseline measures of disease activity, organ damage, biomarkers and medication use recorded at the time of diagnosis are essential for assisting subsequent monitoring of lack of response to initial treatment. Other general risk factors that practitioners should be mindful of are discussed in **Advice point 5**.

(3.2) Experts also highlighted that, while biological use can be associated with high short-term direct costs, this may be offset by reductions in long-term healthcare utilisation and associated direct and indirect costs.^17^,^19^ Supporting that, a post hoc analysis of the Belimumab International SLE Study – Subcutaneous formulation (BLISS-SC) trial reported significantly lower costs of treating flares in biological-treated patients than those who received non-biological standard therapy.^18^

(3.3) The latest treatment recommendations are to initiate first-line LN therapy with a multiagent regimen.^9 11^ Multiagent regimens should comprise a mycophenolic acid analogue (MPAA) or low-dose intravenous cyclophosphamide (CYC), either a biological (appropriate with either MPAA or CYC) or a CNI (CNI not recommended if CYC is used, owing to a lack of clinical trial data and potential toxicity risks of this combination,^9 11 16^ and GCs (initially via intravenous pulse followed by oral GCs to be tapered as described in **Advice points 12 and 13**).^9 11^

**Advice point 4. Regular monitoring of progress towards the goals of treatment is important so that necessary adjustments can be made promptly to keep the patient’s treatment plan to reduce organ damage on track. According to the T2T strategy, treatment plans should specify treatment goals over a specified time period and be agreed with the patient as part of a shared decision-making process.**

**Advice point 5. Identify risk factors for a poor prognosis, which can indicate that a patient may require a more intensive treatment regimen to give the best chance of reaching a treatment goal.**

(5.1) The experts highlighted several risk factors for poor prognosis among patients with SLE based on their clinical experiences and the literature. These included: major organ involvement (eg, kidney, cardiovascular system); presence of comorbidities (such as antiphospholipid syndrome (APS), obesity and hypertension); lifestyle factors such as smoking^20^; male sex or patients of non-white ethnicity; delayed diagnosis or treatment; and socioeconomic factors (eg, low socioeconomic status, limited access to care, cultural barriers).

Specific risk factors for LN development in patients with SLE include younger age (<26), male sex and serological activity at SLE diagnosis.^21^ In cases of multiple organ involvement, practitioners should prioritise treatment of those organs at most risk of accruing irreversible damage. Almost all patients with SLE with haematuria or proteinuria have chronic kidney disease (regardless of whether estimated glomerular filtration rate (eGFR) is impaired) and should be considered at risk of progression.^9^

(5.2) Chronic exposure to GCs (see **Advice points 12–13**), poor medication adherence and accrual of organ damage over time can also be indicative of poor prognosis.

**Advice point 6. Be proactive and avoid unnecessary delays in treatment adjustments when inadequate responses are observed, particularly in patients at risk of poor outcomes.**

(6.1) A practitioner’s own experience plays an important role in the interpretation of early signs of clinical improvement, even if remission or low disease activity targets have not yet been achieved. In such cases, the carefully balanced practitioners’ decisions are crucial for the avoidance of premature treatment changes.

(6.2) A time frame of 6 months may be used to monitor treatment responses after the introduction of a new therapy, although experts acknowledged that this time point is only indicative. Due to disease heterogeneity, timing of treatment responses can vary, and sufficient time should be allowed to observe any treatment responses before adjustments are made.

(6.3) Knowledge of the mode of action of the various medications and the timeframe within which overt clinical responses may be expected is crucial and may guide the need for bridging GC regimens to quickly dampen inflammation and achieve treatment targets, until treatment effects have been established.

(6.4) Non-responses can be considered as either primary (no adequate response after completion of initial therapy) or secondary (an initial response followed by failure to sustain this). For primary non-responses, practitioners may consider changing to a treatment with a different mechanism of action, while secondary non-responses may lead to a reintroduction of the same first-line therapy.

**Advice point 7.** **Encourage patient education and activation, implementing shared decision-making to engage patients and other relevant healthcare professionals in the management of SLE. This is important to ensure patients are aligned with healthcare professionals on the goals and the management plan for their disease and can promote treatment adherence, increasing the likelihood of achieving treatment goals.**

(7.1) Experts highlighted several topics that practitioners are encouraged to discuss with their patients. These include the explanation of GC side effects and alternatives to their use, so that patients understand the need to limit long-term exposure to EULAR recommendation of ≤5 mg/day.^8^ As patients can sometimes be reluctant to reduce their GC dose for fear of symptom worsening, education around the rationale behind limiting long-term GC exposure is important—transfer of this knowledge to patients is the care providers’ responsibility. Similarly, patients can have different preferences on GC tapering—a shared decision-making approach is important to align patient and clinician expectations around the timing and procedure of tapering.

(7.2) Patient education is an important prerequisite for effectively implementing shared decision-making, as advocated by EULAR recommendations for the non-pharmacological management of SLE.^22^ Patients should have a good understanding of their disease, its potential complications and comorbidities and the range of treatment options, thereby providing them with the terminology and knowledge to effectively communicate changes in their disease with practitioners. Patient education should be carried out with empathy and take into account patient preferences on treatment where possible (eg, oral, intravenous or subcutaneous administration).^22^ Of particular importance is ensuring that patients take ownership of the need for continual adherence to their treatments to maximise their effectiveness and improve the chances of positive outcomes. Patients should be made aware that non-adherence is a widespread issue in SLE treatment; physicians should emphasise that this is a part of normal patient education, to ensure the strong messaging around the importance of adherence is not taken personally.

(7.3) It is important to discuss family planning and provide information regarding the teratogenic effects of some treatments.^23 24^ Practitioners are advised to consult specific taskforce recommendations for use of antirheumatic drugs in reproduction, pregnancy and lactation in SLE, such as the recently updated recommendations from EULAR.^25^ The EULAR taskforce notes that the following SLE treatments can be used during pregnancy: azathioprine, belimumab, cyclosporine, hydroxychloroquine, rituximab and tacrolimus.^25^

### Monitoring achievement of targets

**Advice point 8. In addition to general monitoring of disease activity with SLEDAI-2K or PGA, a patient’s individual manifestations should be considered to identify specific aspects of their disease that would benefit from closer monitoring.**

(8.1) Regular evaluation of serological activity, usually by means of complement and anti-double-stranded DNA (dsDNA) levels, is recommended as a part of overall assessments of disease activity. For patients with SLE and LN, proteinuria and eGFR should be evaluated to monitor progression of kidney disease, with the ultimate goal of avoiding end-stage kidney disease.

(8.2) In addition to a global index, for example the SLEDAI^8 26^ or the British Isles Lupus Assessment Group (BILAG) index scores,^27^ practitioners are encouraged to measure disease activity in single organs, such as the use of swollen and tender joint counts for joint manifestations or the Cutaneous Lupus Erythematosus Disease Area and Severity Index^28^ (CLASI) for skin disease. If these tools are not feasible due to time constraints, patient photographs before and after treatment may prove valuable.

(8.3) Pregnant patients with SLE should be monitored more intensively, together with obstetrics specialists in high-risk pregnancies and potentially a haematology consultation (eg, coagulation specialists) if the patient has positive antiphospholipid antibodies or APS.

**Advice point 9. Monitor changes in organ damage over time, even in patients with mild disease, by use of the Systemic Lupus International Collaborating Clinics/American College of Rheumatology (SLICC/ACR) Damage Index (SDI) at the first visit, and then annually.**

(9.1) Irreversible organ damage is a predictor of death and morbidity in SLE, with each additional SDI point being associated with significantly increased risks of mortality.^29^ Similarly, as even one unit of increase in the Safety of Estrogens in Lupus Erythematosus National Assessment (SELENA)-SLEDAI disease activity index has been associated with increased risk of subsequent damage,^30^ it is important to monitor patients annually for signs of organ damage progression using the SDI.

(9.2) National Institutes of Health (NIH) activity and chronicity indices scored from repeat kidney biopsies can be used to inform responses to treatment, remission status and prognosis for poor long-term kidney outcomes by using definitions recently proposed by the Lupus Nephritis Trials Network.^9 31^ The 2025 EULAR recommendations for LN particularly advise repeat biopsies, especially in cases of clinical uncertainty, to help evaluate treatment responses, worsening of kidney-specific laboratory tests and potential withdrawal of IS treatment.^9^

**Advice point 10. While some treatments do improve patient-reported outcome measures (PROMs), more evidence is needed regarding the impact of their use on outcomes and to clarify how monitoring changes in PROMs over time should inform treatment decisions.**

(10.1) Most experts reported that they do not use PROMs routinely in their practice, outside of clinical studies, predominantly due to time constraints. There are many PROMs for SLE, translated and validated in several languages, and other non-SLE-specific PROMs that are used in clinical trials.

### Optimisation of therapeutic strategy to prevent flares while facilitating GC dose reductions

**Advice point 11. Practitioners should strive to prevent flares, as each one, regardless of severity, is associated with an increased risk of organ damage accrual.**^32^ **Practitioners can consider SLE flares to be the emergence of new signs or symptoms, worsening of existing symptoms or increases in disease activity that necessitate, or lead to consideration of, a change or escalation in therapy (eg, new organ involvement, or increased severity of known manifestations), consistent with the International Flare Consensus Initiative.**^33^

(11.1) Approaches to prevent flares in clinical practice include optimising antimalarial and conventional IS doses and biological use; slow tapering of GCs and IS and not removing treatments too early; regular screening and monitoring of biomarkers, such as anti-dsDNA and complement, to predict or identify early signs of flare and allow for appropriate action to be taken as early as possible. We suggest that antimalarial agents can be continued long-term if tolerable and in the absence of contraindications.

In LN approaches to flare prevention include checking for proteinuria and haematuria, as well as eGFR to detect kidney flares or *de novo* kidney involvement, while monitoring serum creatinine and eGFR that reflect kidney function.

(11.2) The intensity of an SLE flare can be assessed using tools such as the SELENA-SLEDAI Flare Index (see box 2)^26^; however, experts appreciate that such tools are not always feasible for use in routine practice and are more commonly used within clinical studies.

Box 2 **Safety of Estrogens in Lupus Erythematosus National Assessment-Systemic Lupus Erythematosus Disease Activity Index (SELENA-SLEDAI) flare index^26^**

**Severe flare:**

**Advice point 12. Achieving the recommended GC target of ≤5 mg/day prednisone-equivalent within the shortest feasible time period (ideally within 6 months) can help reduce GC-associated toxicities, such as irreversible organ damage.** 8 10

(12.1) The approach taken towards reducing GC doses should account for a patient’s disease history and duration, be implemented as part of a shared decision-making process and cannot follow strict guidelines. Adjustments should consider an individual patient’s manifestations and organ involvement, disease severity, comorbidities, history of flares, cumulative GC exposure, patient preferences and needs, GC toxicity and risks and treatment adherence.

- For newly diagnosed patients, or those with a short duration of disease, practitioners should discuss alternatives to sustained GC use in these patients, such as the earlier introduction of conventional ISs and/or biologics. In support of this, clinical and real-world studies have reported improved rates of GC tapering with biologics and some ISs, such as mycophenolate mofetil.^34^,^39^
- For patients with severe disease or major organ involvement (such as active LN), practitioners can consider multiagent therapy (together with GC), per international recommendations.^8 9 11^ Practitioners can also reduce exposure to high doses of systemic GCs by considering the use of intravenous methylprednisolone pulses prior to commencing oral GC therapy.^9^

(12.2) Despite several tapering regimens reported, with little evidence available, experts noted that they typically aim to reduce doses to ≤5 mg/day within 4–6 months (for SLE with or without LN).^40 41^ After this, GC tapering can proceed more slowly.

(12.3) For patients with established SLE on chronic GC treatment, withdrawal can increase the risk of flares—dose reductions should be gradual and carefully monitored, with the ultimate aim to withdraw GCs entirely, if feasible.

(12.4) Practitioners should also be mindful that patients can respond differently to GC tapering; the overall objective is to taper GCs in a manner that minimises the risk of flares or new SLE organ involvement. As previously mentioned, it is important to be especially vigilant when monitoring GC withdrawal in patients who have been receiving GCs long-term.

**Advice point 13. Practitioners should consider that even achievement of DORIS remission should not prompt the termination of efforts to further reduce the GC dose. It can be helpful to view GC tapering as a two-step process; first, to reach 5 mg/day of a prednisone equivalent, and then to continue tapering below 5 mg/day of a prednisone equivalent in an individualised manner until full withdrawal, whenever possible. In LN, further dose reduction below 5 mg/day of a prednisone equivalent is recommended if patients have a sustained complete response.**

## Discussion

SLE is characterised by a high level of clinical heterogeneity, both in the breadth of potential organ manifestations, severity and the ‘relapse-remitting’ fluctuating disease course.^1 2^ As such, a proactive approach with regular monitoring^42^ is essential to guide decisions, prevent disease flares and identify early changes in disease activity and organ involvement, together enabling timely adjustments to treatment and reducing risks of irreversible damage. These complexities can pose significant challenges for practitioners in determining the most appropriate management strategies.

The EULAR recommendations for SLE management provide practitioners with a comprehensive framework that may help their patients achieve the best possible outcomes.^8^ Central to these recommendations is the need to adopt the T2T strategy by aiming for clinically meaningful targets,^42^ such as DORIS remission or LLDAS, and the need to limit cumulative GC exposure to reduce the risk of organ damage progression and other toxicities associated with GC use.^8^

An inherent problem of all management recommendations is that they cannot capture all aspects of routine clinical care. This leads to challenges faced by practitioners when trying to implement the EULAR recommendations in daily practice. To address this, the practical and expert-driven advice provided in this manuscript may help the implementation of the EULAR recommendations effectively and efficiently within routine daily practice. Practitioners must remain mindful that each patient’s disease course is unique and should strive to individualise treatment plans, while ensuring that the patient receives sufficient education on their disease and treatment options.

Areas of strong agreement among the SLE experts included the importance of T2T for long-term disease control and organ protection. There was also agreement on the necessity of frequent monitoring, with a recommended interval of approximately every 3 months for disease activity evaluation (though this may need to be more or less frequent depending on severity), and at least annual evaluations for organ damage. This regular assessment allows practitioners to detect changes in a patient’s disease and proactively escalate or modify treatments before organ damage occurs. The experts also highlighted a growing trend towards earlier consideration of biological therapies, such as belimumab and anifrolumab, which have demonstrated efficacy in several randomised clinical trials and real-world studies^43^,^47^ and may facilitate GC dose reductions.^36 45^ There was strong agreement for reducing GCs to ≤5 mg/day at the earliest clinically feasible opportunity, and then slowly tapering towards the ultimate goal of full withdrawal, if feasible.

There are several barriers to implementing the EULAR recommendations and a T2T approach in real-world practice that have yet to be fully addressed. For example, practitioners are often limited by time constraints during patient visits, have inadequate resources, access only to certain treatments or limited experience in SLE treatment, and clinical inertia can discourage practitioners from incorporating the latest recommendations in their clinic. Although systemic barriers such as medication access and reimbursement may take time to resolve, the practical advice provided in this manuscript may help to indirectly address some barriers by encouraging practitioners to most effectively and efficiently implement the latest clinical recommendations within the common constraints of real-world SLE practice.

Regarding the future treatment landscape, experts noted that there is a need for identification of more SLE-specific biomarkers, reliable predictors of prognosis, indicators of ‘partial treatment response’ and more evidence of the use of PROMs in clinical practice to help guide treatment. Future research in these areas, such as improved disease activity biomarkers and more robust clinical tools, could provide practitioners with more confidence when tailoring therapies to each individual patient, enabling them to escalate or switch treatments appropriately within the wider framework of the EULAR recommendations. Several other frameworks and guidelines have become available in recent years to help optimise treatment in daily practice, including the SLE and LN disease modification framework,^5^ a 2025 framework for implementing T2T in routine care,^7^ ACR 2024, EULAR 2025 and Kidney Disease: Improving Global Outcomes (KDIGO) 2024 guidelines for LN,^9 11 16^ EULAR recommendations for use of antirheumatic drugs in pregnancy^25^ and EULAR non-pharmacological recommendations for SLE and systemic sclerosis.^22^ Similarly, comprehensive guidance is available on screening and prophylaxis of chronic or opportunistic infections and the management of cytopenias in SLE.^48 49^

In conclusion, the complexity and heterogeneity of SLE require a proactive, patient-centred management approach. The EULAR recommendations provide an important framework for improving outcomes by focusing on individualised treatment and emphasising the avoidance of GC toxicity. Practitioners who incorporate the EULAR recommendations into routine patient visits, which the practical advice we provide in this manuscript aims to facilitate, have the best chance of helping patients achieve sustained remission or low disease activity. Although barriers persist, especially in terms of time constraints and dearth of biomarkers, we hope that the advice we offer can provide practitioners with strategies that help overcome these challenges. Ongoing research and collaboration across disciplines will remain important to advancing SLE care. By striving for standardised goals such as remission or low disease activity and continuing to evolve the tools and treatments available, and further recognising the benefits of earlier effective intervention, we can significantly improve the lives of our patients living with SLE.

## Supplementary material

10.1136/rmdopen-2025-006210 online supplemental file 1

## Data Availability

Data sharing not applicable as no data sets generated and/or analysed for this study.
