# Supplementary material for: Practical insights for the clinical implementation of the EULAR recommendations for patients with systemic lupus erythematosus
Source: RMD Open. 2025 Dec 31;11(4):e006210. doi: 10.1136/rmdopen-2025-006210 (PMC12766831; doi:10.1136/rmdopen-2025-006210)
Supplement: online supplemental file 1 [file rmdopen-11-4-s001.pdf]

Supplementary Materials

Supplementary Material 1. Online Survey Questions

Section 1. EULAR recommendations on early diagnosis and treating-to-target (T2T)

Early SLE diagnosis (including serological assessment), regular screening for organ involvement (especially nephritis), prompt initiation of treatment aiming at remission (or low disease activity if this is not possible), and strict adherence to treatment are essential to prevent flares and organ damage, improve prognosis and enhance quality of life.

1. What strategies do you currently employ to integrate T2T within your clinical practice, if any?
2. What do you perceive are the barriers to implementing T2T in clinical practice? Please provide any thoughts on how these may be overcome
3. What tools or approaches do you use in your practice to help diagnose SLE as early as possible?
4. How often do you measure disease activity and organ damage in routine practice? Which tool(s) do you use?
5. Do you routinely use patient-reported outcome measures (PROMs)? If so, please specify which PROMs you use?
6. How do you define remission or low disease activity state in your practice, and approximately how often do patients reach these goals (% of remission/% LDA)?
7. How do you define SLE relapse? What strategies do you use to help prevent relapses or flares?
8. In your experience, what are the key risk factors for poor prognosis or long-term outcomes that may inform the management of your patients?

Section 2. EULAR recommendations on glucocorticoid (GC) use

Glucocorticoids, if needed, are dosed based on the type and severity of organ involvement (**2b/C**), and should be reduced to maintenance dose of ≤5 mg/day (prednisone equivalent) (**2a/B**) and, when possible, withdrawn; in patients with moderate-to-severe disease, pulses of intravenous methylprednisolone (125–1000 mg per day, for 1–3 days) (**3b/C**) can be considered.

1. What strategies do you currently employ to help limit unnecessary GC exposure?
2. What do you find are the major barriers to being able to reduce GCs to <5 mg/day for most patients?
3. When tapering GCs, is there a preferred regimen that you use to help achieve a dose <5 mg/day? Is this regimen informed primarily by the patient's disease severity, manifestations, or other factors?
4. Over what average timeframe do you aim to taper GCs to <5 mg/day?

Section 3. EULAR recommendations on immunosuppressants (IS) and biologic use

In patients not responding to hydroxychloroquine (alone or in combination with glucocorticoids) or patients unable to reduce glucocorticoids below doses acceptable for chronic use, addition of immunomodulating/immunosuppressive agents (eg, methotrexate (**1b/B**), azathioprine (**2b/C**) or mycophenolate (**2a/B**)) and/or biological agents (eg, belimumab (**1a/A**) or anifrolumab (**1a/A**)) should be considered.

1. What strategies do you currently employ to identify which patients are suitable for IS or biologics?
2. What are the barriers to implementing the EULAR recommendations on biologic use in clinical practice? Please provide any suggestions on how they may be overcome
3. Do you typically use biologics or conventional IS first, or concomitant treatment? What are the major factors that you consider when deciding to prescribe biologics earlier (after HCQ and GC with or without IS)? Is this dependent on the patient's individual disease presentation?
4. Which characteristics should a patient have to start a biologic after HCQ and GC (with or without IS)?
5. Do you taper IS treatments after any specific period of time following response to therapy?
